# The optic nerve head in glaucoma

**Published:** 2012

**Authors:** Rupert RA Bourne

**Affiliations:** Consultant ophthalmic surgeon and glaucoma specialist: Hinchingbrooke, Moorfields, and Addenbrookes Hospitals, UK, and Professor of Ophthalmology, Anglia Ruskin University, UK.

**Figure:**
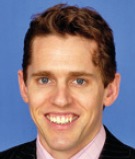
Rupert RA Bourne

All types of glaucoma involve glaucomatous optic neuropathy. The key to detection and management of glaucoma is understanding how to examine the optic nerve head (ONH).

This article addresses the following issues:

- how to examine the ONH
- normal characteristics of the ONH
- characteristics of a glaucomatous ONH
- how to tell if the glaucomatous optic neuropathy is getting worse

The ONH can be examined using a direct ophthalmoscope, an indirect ophthalmoscope, or a posterior pole lens with a slit lamp.

Many types of health professional can assess the ONH accurately after having appropriate training. Dilating the pupil makes this easier and will improve the accuracy of the examination, regardless of which instrument is used. Where the equipment is available, more sophisticated techniques such as scanning laser polarimetry, confocal scanning laser ophthalmoscopy, and ocular coherence tomography can also be used to complement the clinical examination of the ONH and provide quantitative measurements.

The time available to view the ONH is often short as the examination is uncomfortable for the patient. It is therefore essential that the examiner has a strategy for making the observations needed to distinguish a glaucomatous ONH from a normal ONH.

Before you start, you should first be able to recognise the characteristics of both a normal and a glaucomatous ONH, and be able to look for additional signs that could indicate a glaucomatous ONH.

## Characteristics of the normal ONH (Figure 1)

The ONH, or optic disc, is a round/oval ‘plughole’ down which more than a million retinal nerve fibres descend through a sieve-like sheet known as the lamina cribrosa. The retinal nerve fibres are then bundled together behind the eye to form the optic nerve which then continues towards the brain.

The retinal nerve fibres are spread unevenly across the surface of the retina in a thin layer which has a ‘feathery’ appearance, best seen immediately above and below the disc (Figure 2).

**Figure 1:**
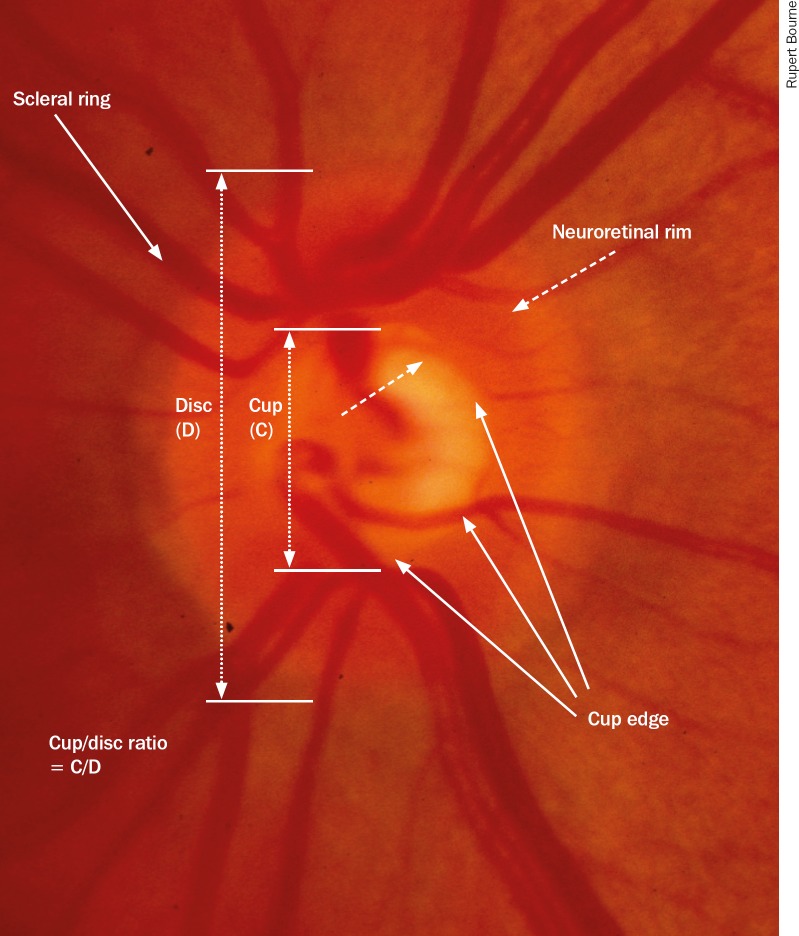
Normal optic nerve head

As the nerve fibres approach the edge of the disc they pour over the **scleral** ring (which marks the edge of the disc) and then down its inner surface. The dense packing of nerve fibres just inside the **scleral** ring is visualised as the neuroretinal rim. The cup is the area central to the neuroretinal rim. The cup edge (where it meets the neuroretinal rim) is best seen by the bend in small and medium-sized blood vessels as they leave, or descend into, the cup.

Most normal discs are more vertically oval and their cup more horizontally oval.

In addition, most (but not all) normal ONHs obey the ‘ISNT’ rule: the Inferior (lower) rim is usually thicker than the Superior (upper) rim, which is thicker than the Nasal rim (inner, nearest the nose). The Temporal rim (outer, nearest the temple) is the thinnest.

**Figure 2:**
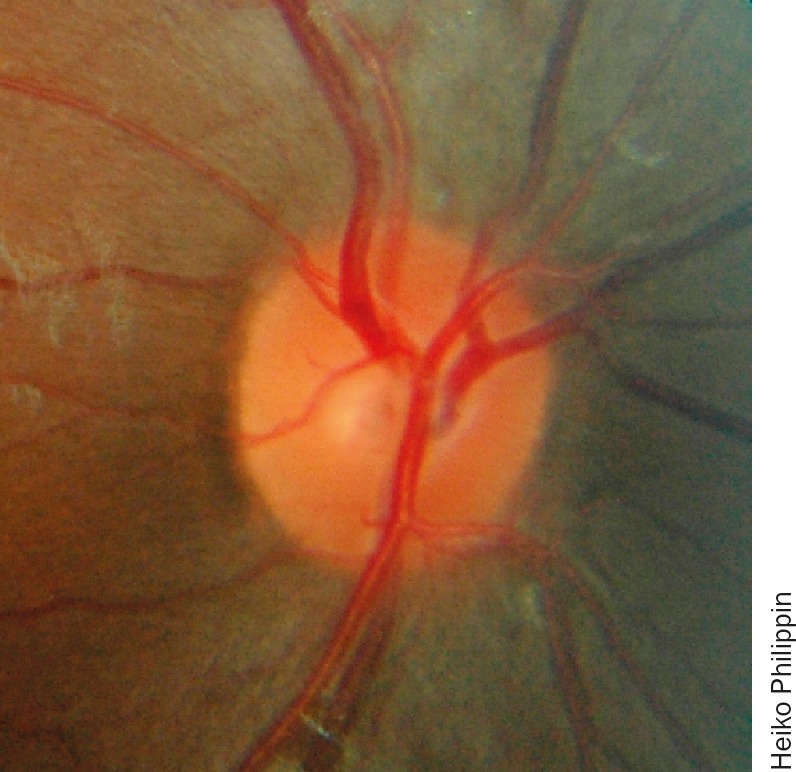
Normal optic nerve head of a young African patient

**Figure:**
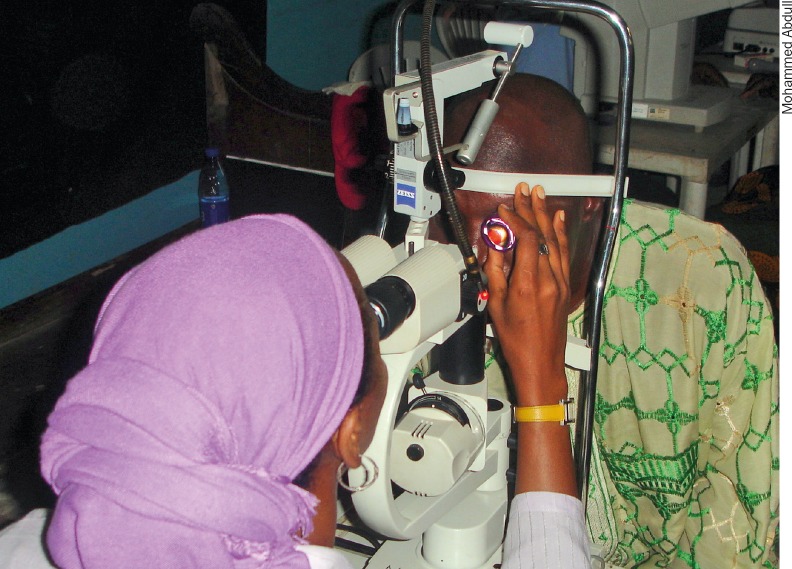
Examining the optic nerve using a slit lamp and posterior pole lens. This offers a stereo view, high magnification, and good illumination. NIGERIA

## Characteristics of a glaucomatous ONH

**Figure 3:**
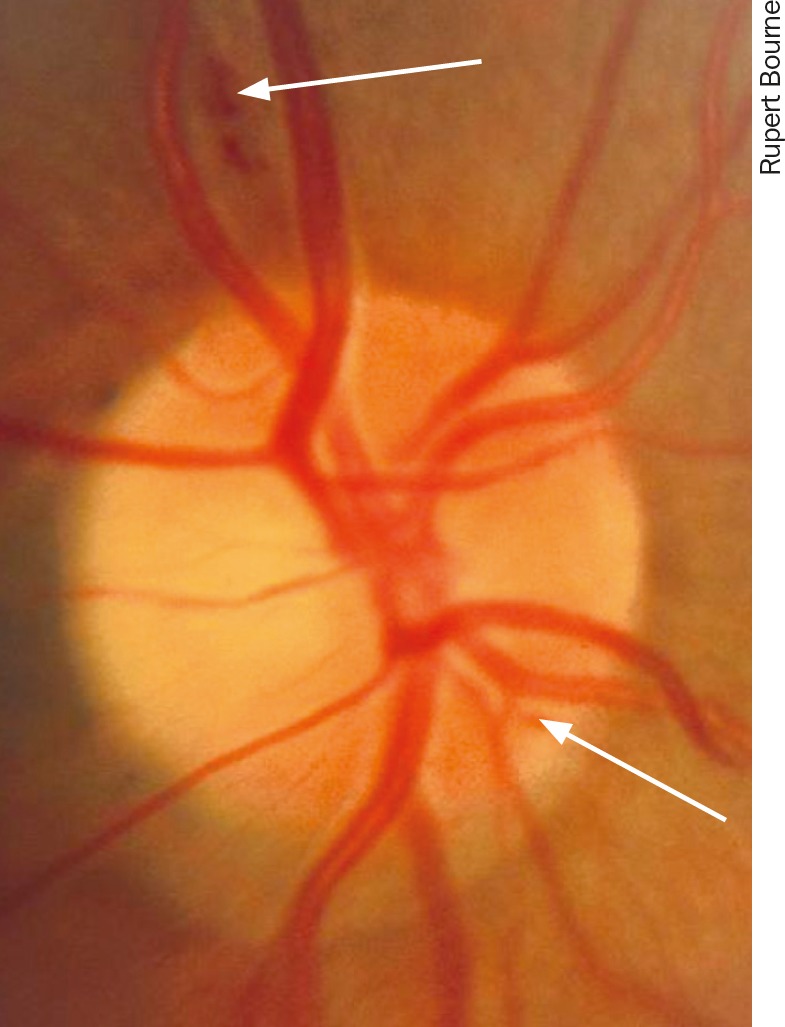
Glaucomatous optic neuropathy: splinter haemorrhages

**Figure 4:**
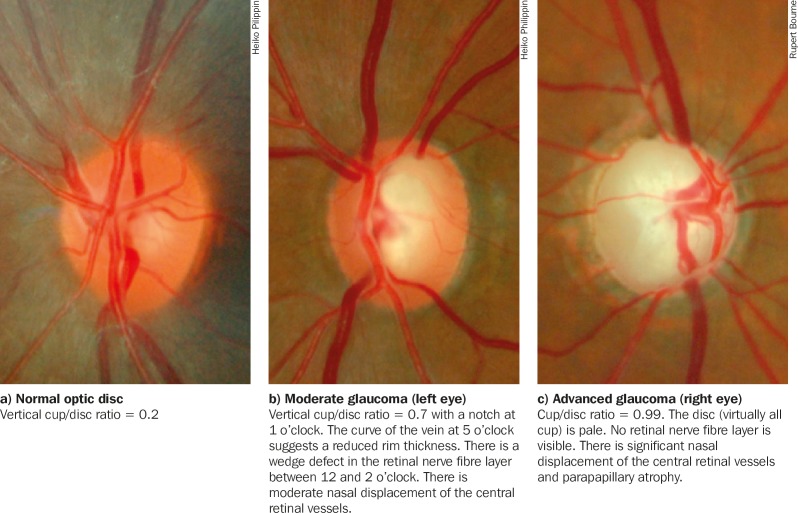
Normal optic disc (a) and glaucomatous optic nerve heads of two patients with different severities of glaucoma

**Figure 5:**
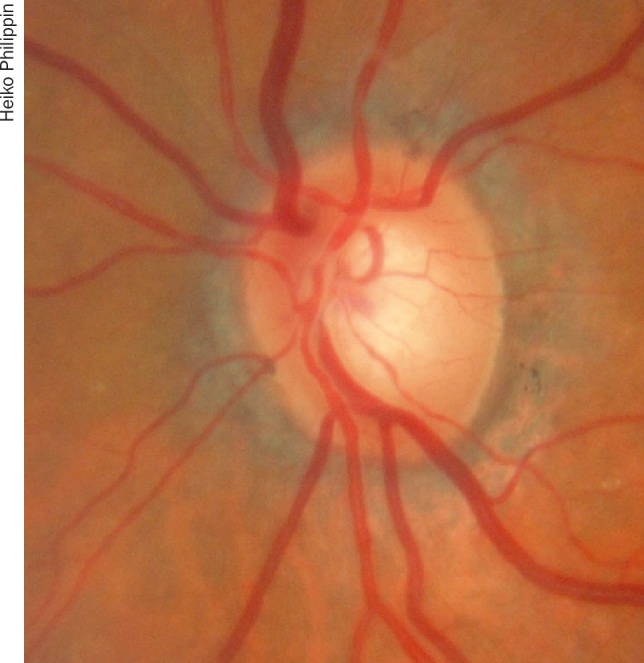
(left) Glaucomatous optic nerve head of a patient with pseudoexfoliation glaucoma (PXFG). The demarcation of the cup by the blood vessels differs from the margin between the pallor of the base of the cup and the surrounding pinker colour between this and the disc edge. Focussing on the colour difference is misleading. One should judge the edge of the rim by the change in direction of the small and medium-sized vessels which, in this case, indicates a thinner rim than might be suspected by the colour difference

**Figure 6:**
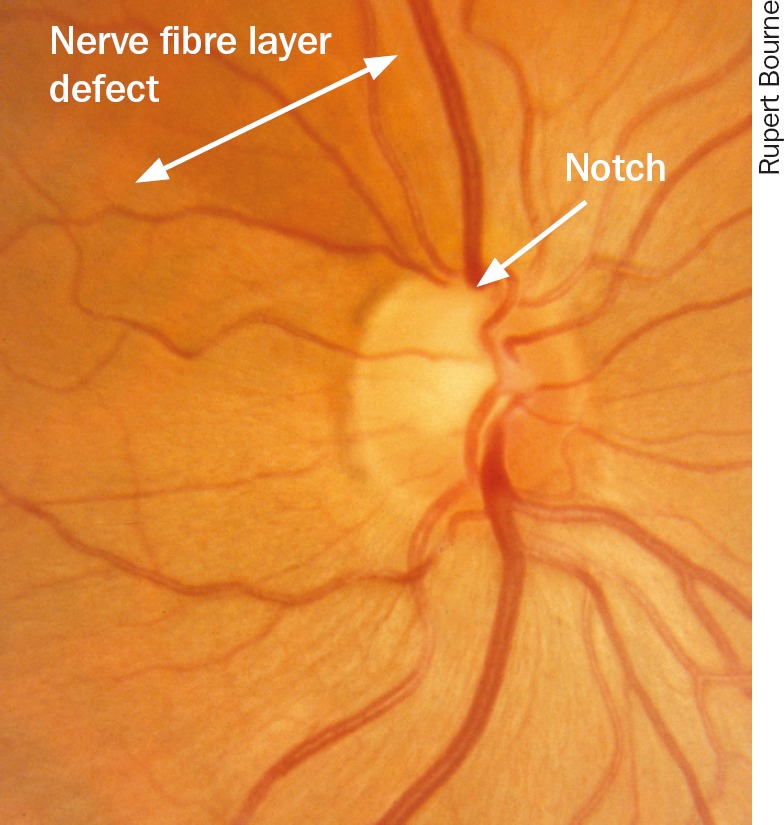
Glaucomatous optic neuropathy: focal enlargement of cup (notch) and nerve fibre layer defect

- Generalised/focal enlargement of the cup. (Note that the cup always appears smaller when viewed monoscopically than in stereo)
- Disc haemorrhage (within one disc diameter of ONH) (Figure 3)
- Thinning of neuroretinal rim (usually at the superior and inferior poles) (e.g. Figures 4 and 5)
- Asymmetry of cupping between patient's eyes
- Loss of nerve fibre layer (Figure 6).

## Additional signs which should heighten suspicion of a glaucomatous ONH

- Cup/disc ratio (CDR) ≥0.7. A measurement of CDR alone is insufficient and may be misleading as small discs will have smaller cups and hence a smaller CDR. It is important, therefore, to document disc size by measuring the vertical height of the disc. In most populations, only 5% of people with no glaucoma will have a CDR of ≥0.7.
- Rim does not obey the ISNT rule
- Presence of parapapillary atrophy (more common in glaucomatous eyes).

## Strategy: distinguishing a glaucomatous ONH from a normal ONH

1. Dilate pupils, if possible and safe to do so.
2. Identify the disc edge and cup edge, and identify the rim.
3. Does the rim thickness obey the ISNT rule?
4. Is there a haemorrhage?
5. Measure the vertical height of the ONH*
6. Estimate the vertical CDR.
7. Examine the retinal nerve fibre layer (using green light).*
8. Draw an annotated diagram of the ONH.

*This may only be possible with a slit lamp and posterior pole lens.

## Is the glaucomatous optic neuropathy worsening or progressing? (Figure 7)

The appearance of any of the features of a glaucomatous ONH, or the exacerbation of these features compared to a previous record, is indicative of a progression/ worsening of the disease.

**Figure 7:**
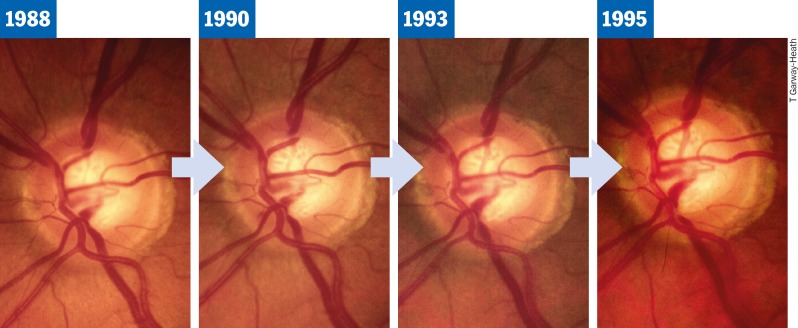
An example of progression of glaucomatous optic neuropathy (left eye) over seven years.

Disc haemorrhages may be present for two weeks to three months and are an important prognostic sign of progression. An accurate record requires careful observation and a detailed drawing, and photographic documentation (preferably stereophotography) is highly recommended.

Other imaging devices offer progression analyses, but these are not a surrogate for a detailed clinical examination.

Progressive worsening of the visual fields should correlate with structural changes at the ONH.

**Pitfalls and pearls**

- The hallmark of glaucomatous optic neuropathy is excavation of the neuroretinal rim
- Advanced glaucomatous ONH can result in a pale optic disc, but disc pallor should also raise suspicion of another cause such as optic atrophy
- A colour difference should not be used to distinguish the cup edge; change in direction of the blood vessels is a more reliable indicator (Figure 5)
- The optic disc abnormality should correlate with the visual field defect. Where this is not the case, further investigations (e.g. CT/MRI scan) may be indicated
- The size of the cup always appears smaller when viewed monoscopically rather than steroscopically.
